# Metformin Strongly Affects Gut Microbiome Composition in High-Fat Diet-Induced Type 2 Diabetes Mouse Model of Both Sexes

**DOI:** 10.3389/fendo.2021.626359

**Published:** 2021-03-19

**Authors:** Laila Silamiķele, Ivars Silamiķelis, Monta Ustinova, Zane Kalniņa, Ilze Elbere, Ramona Petrovska, Ineta Kalniņa, Jānis Kloviņš

**Affiliations:** Latvian Biomedical Research and Study Centre, Riga, Latvia

**Keywords:** metformin, microbiome, metagenome, high-fat diet, C57BL/6N

## Abstract

Effects of metformin, the first-line drug for type 2 diabetes therapy, on gut microbiome composition in type 2 diabetes have been described in various studies both in human subjects and animals. However, the details of the molecular mechanisms of metformin action have not been fully understood. Moreover, there is a significant lack of information on how metformin affects gut microbiome composition in female mouse models, depending on sex and metabolic status in well controlled experimental setting. Our study aimed to examine metformin-induced alterations in gut microbiome diversity, composition, and functional implications of high-fat diet-induced type 2 diabetes mouse model, using, for the first time in mice study, the shotgun metagenomic sequencing that allows estimation of microorganisms at species level. We also employed a randomized block, factorial study design, and including 24 experimental units allocated to 8 treatment groups to systematically evaluate the effect of sex and metabolic status on metformin interaction with microbiome. We used DNA obtained from fecal samples representing gut microbiome before and after ten weeks-long metformin treatment. We identified 100 metformin-related differentially abundant species in high-fat diet-fed mice before and after the treatment, with most of the species relative abundances increased. In contrast, no significant changes were observed in control diet-fed mice. Functional analysis targeted to carbohydrate, lipid, and amino acid metabolism pathways revealed 14 significantly altered hierarchies. We also observed sex-specific differences in response to metformin treatment. Males experienced more pronounced changes in metabolic markers, while in females the extent of changes in gut microbiome representatives was more marked, indicated by 53 differentially abundant species with more remarkable Log fold changes compared to the combined-sex analysis. The same pattern manifested regarding the functional analysis, where we discovered 5 significantly affected hierarchies in female groups but not in males. Our results suggest that both sexes of animals should be included in future studies focusing on metformin effects on the gut microbiome.

## Introduction

Metformin is the first-line therapy for the treatment of type 2 diabetes (T2D). According to the American Diabetes Association and European Association for the study of Diabetes guidelines, it is the preferred option for initiating glucose-lowering due to its efficacy, safety, tolerability, and low cost (1)⁠⁠. The molecular mechanism of action of metformin, however, remains unclear.

⁠It is generally considered that metformin’s antihyperglycemic activity is mainly due to the reduced hepatic glucose production, thus activating pathways independent of AMP-activated protein kinases (AMPKs) (2)⁠⁠. There is an increasing evidence that metformin’s action mechanism is associated with physiological processes in the gastrointestinal tract. For example, a more pronounced effect of metformin can be observed when the drug is administered orally than intravenously at an equivalent dose (3)⁠. It has been estimated that 20-30% of people receiving metformin therapy develop gastrointestinal side effects, with approximately 5% being unable to tolerate metformin at all (4)⁠⁠⁠. Metformin accumulates in gastrointestinal tissues (5)⁠⁠; for example, it is 30-300 times more concentrated in the small intestine than in plasma, and 30-50% of the drug reaches the colon and is eliminated with feces (6)⁠⁠.⁠

Studies in humans have convincingly shown that metformin specifically alters gut microbiome both in T2D patients (7–9)⁠⁠⁠ and healthy subjects (10, 11). A multi-country cross-sectional study employing metformin-untreated T2D patients, metformin-treated T2D patients, and non-diabetic controls has shown depletion of butyrate producers such as *Roseburia*, *Subdoligranulum*, and butyrate-producing *Clostridiales* spp. in the metformin-untreated T2D subset; in turn, metformin treatment significantly increased the abundance of *Escherichia* spp. and decreased the abundance of *Intestinibacter* and, when analyzed functionally, significantly augmented butyrate and propionate production potential (7)⁠. These results have been approved further by a longitudinal double-blind study on individuals with treatment-naive T2D (8)⁠. Another direction of metformin’s effects on the gut microbiome is the increased abundance of the mucin-degrading *Akkermansia muciniphila* (12). Studies in healthy individuals have reported an increase of *Escherichia/Shigella* and a decrease in the abundance of *Intestinibacter* spp. and *Clostridium* spp. 10,11). It has been shown that metformin exerts its effects on the microbiome already in the first 24 hours (10)⁠ and that the alterations in the gut microbiome could be related to the metformin-induced immune response (13)⁠.

A number of studies that have specifically targeted metformin effects in rodents have used 16S rRNA sequencing and have identified that metformin modifies the metabolic profile of high-fat diet-fed animals accompanied with changes in the microbiome (14–16)⁠. Reduced abundances of *Akkermansia* and *Alistipes* and the increases in the proportions of *Anaerotruncus*, *Lactococcus*, *Parabacteroides*, *Odoribacter*, *Lawsonia*, *Blautia*, and *Lactonifactor* have been reported in high-fat diet-fed mice, which were countered by metformin treatment (17)⁠. The gut microbiome of high-fat diet-fed mice is dominated by *Firmicutes*, in contrast to mice fed the regular diet in which *Bacteroidetes* prevail. Lee et al. have demonstrated that metformin treatment increases *Bacteroidetes* abundance to a level similar to that of the control mice and changes the abundances of *Bacteroidaceae*, *Verrucomicrobiaceae*, *Clostridiales family XIII, incertae sedis*, and *Akkermansia muciniphila* (18). Metformin exerts changes in the gut microbiome of healthy mice also by increasing the abundances of *Rikenellaceae*, *Ruminococcaceae*, *Verrucomicrobiaceae*, *Alistipes* spp., *Akkermansia* spp., and *Clostridium* spp. (18). A study on the modulation of the gut microbiome in a high-fat diet-fed male rat model in response to metformin treatment has shown increased abundances of *Blautia*, *Bacteroides*, *Butyricoccus*, *Phascolarctobacterium*, *Parasutterella*, *Akkermansia*, *Prevotella*, *Lactobacillus*, and *Allobaculum* (16).

A recent review on the effects of metformin on the gut microbiome in the context of obesity and T2D has summarized the differences in the results obtained in both human and animal studies (19)⁠⁠ showing that certain unclarities still remain, for example, the directions of changes in response to metformin treatment in the abundances of *Prevotella* and *Lactobacillus* differ in various studies.

This study’s main objective was to evaluate the effect of metformin on the mice microbiome at the strain level that has not been performed before and understand the impact of other factors on this process. We hypothesize that metabolic status at the time of intervention and the sex of the animals have a strong influence on the metformin actions. All of the published animal studies focusing on metformin effects on T2D-related gut microbiome have exploited the 16S rRNA gene sequencing strategy; therefore, as far as we are aware, this is the first study reporting the results of such analysis obtained by shotgun metagenomic sequencing. This approach allows the best taxonomic resolution. Furthermore, our research provides novel information on potential functions by which the gut microbiome contributes to the antidiabetic effects of metformin addressed in each of the sexes separately.

## Materials and Methods

### Study Design

We designed this study as a randomized block experiment comprised of three blocks with a three-way factorial treatment arrangement where factors of interest are T2D status induced by high-fat diet (HFD) or control diet (CD) feeding, sex, and metformin therapy status, forming eight different treatment groups – HFD_M_Met-, HFD_F_Met-, HFD_M_Met+, HFD_F_Met+, CD_M_Met-, CD_F_Met-, CD_M_Met+, and CD_F_Met+ (**Supplementary Figure 1**). Study’s sample size was determined by the Resource Equation method, appropriate for complex designs (20)⁠⁠. In each of the eight treatment groups, we included three experimental units, 24 in total. As the experimental unit was a cage with three animals – the total number of animals involved in the experiment was 72.

After the adaptation week, all experimental units of the same block were randomly assigned to HFD- or CD-fed groups so that each of the treatment groups would consist of animals with similar body weight, and experimental units of both sexes would be represented in the same number in both types of treatment groups. After the induction of T2D manifestations, experimental units were randomly assigned to receive or not to receive metformin treatment providing that the number of experimental units in each of the groups is equal. During all the procedures, treatments, and measurements, as well as sample collection were performed randomly within the same block. Work with each of the blocks was performed on separate days of the week, keeping the interval between interventions in the same block constant. Blinding was applied where appropriate.

### Experimental Animals

Age-matched 4-5-week-old male and female C57BL/6N mice with specific pathogen-free (SPF) status were obtained from the University of Tartu Laboratory Animal Centre.

All the animals were housed in SPF conditions, 23 ± 2 °C, 55% humidity. The light cycle was 12:12 hours, with a light period from 7:00 am to 7:00 pm. All the procedures were performed during the first half of the day in a specially designated procedure room.

Animals were housed in individually ventilated cages (Techniplast) up to three same-sex animals per cage on aspen bedding mixed with ALPHA-dri. All the animals had free access to drinking water. Animals were fed HFD or CD *ad libitum*.

All the cages were enriched with cardboard tunnels, plastic shelters, wooden sticks, and nesting material. For the whole duration of the experiment, animals were observed once a day; if we observed any type of suffering that could not be alleviated, the suffering animal was euthanized by cervical dislocation. During the study humane endpoint was implemented for 13 animals mainly due to male fighting wounds. Thus 59 animals completed the study, however, at the end of the experiment each of the experimental units remained represented by at least one animal.

### Experimental Procedures

After aone to two-weeks long adaptation period during which animals were fed regular chow diet *ad libitum* and received regular drinking water a diet change was initiated. The age of animals at this point was approximately six weeks. All the cages from each of the blocks were randomly assigned to a high-fat diet-fed group or control diet-fed group. Animals were provided with a rodent diet containing 60 kcal% fat (D12492, Research Diets) or rodent diet with 10 kcal% fat (D12450J, Research Diets) *ad libitum*. Both types of diets were sterilized by irradiation. Body weight and food intake (per cage) were measured once a week (see **Supplementary Data** for further details); water intake (per cage) – twice a week.

The cages were changed on a weekly basis. Upon opening the cage, each mouse was immediately transferred to a clean, separate box in which animals were allowed to defecate voluntarily. Each of the animals was weighted. Feces were collected in sterile tubes in three aliquots. Bottles with drinking water were changed two times a week.

Four weeks after starting the assigned diet, we initiated a measurement of blood glucose levels at two weeks interval using an Accu-Chek Performa glucometer (Roche). Blood for regular glucose level measurements was obtained from the saphenous vein by puncturing the vein with a 25G needle. Accu Chek Performa glucometer with Accu Chek test strips was used to measure the glucose level in blood samples. Before the procedure animals were fasted for 6 hours starting from 8:00 am to 2:00 pm. The induction of T2D was evaluated by glucose and insulin level measurements in plasma samples at week 20 after starting the assigned diet. The plasma necessary for the analysis was obtained from blood drawn from the saphenous vein. To estimate the insulin resistance, HOMA-IR index was calculated (by formula $HOMA-IR=\frac{glucose,mmol|L*insulin,mU|L}{180}$) based on fasting plasma glucose and insulin levels determined by the mouse glucose assay and mouse insulin ELISA kit (both from Crystal Chem) at week 20.

Metformin was provided to mice with drinking water. The concentration of metformin was calculated to correspond to 50 mg/kg body mass/day. During the therapy period, all of the bottles, including those of the control group, were changed every day. Metformin was freshly added to the drinking water every day upon water changing. The duration of metformin therapy was ten weeks.

All the animals were sacrificed by cervical dislocation without any other anesthesia, as the effect of other medications would interfere with the study’s aims.

### Microbial DNA Isolation and Shotgun Metagenomic Sequencing

The DNA from fecal samples representing each experimental unit at two timepoints – before and after the metformin treatment (N = 48) was extracted with the FastDNA^®^SPIN Kit for Soil (MP Biomedicals) following manufacturer’s instructions. DNA yield was determined using the Qubit dsDNA HS Assay Kit on the Qubit^®^ Fluorometer 1.0 (Invitrogen Co.). The extracted DNA samples were then diluted to a concentration of 5 ng/μl.

Libraries for metagenomic shotgun sequencing were prepared using MGIEasy Universal DNA Library Prep Kit (MGI Tech Co., Ltd.); the construction of libraries was completed in a single batch. The input of DNA was 200 ng. Library preparation was performed according to the Universal DNA Library Prep Set User Manual and spiked with 1% PhiX. Preparation steps briefly: DNA shearing into 300 bp fragments by S220 focused-ultrasonicator (Covaris) followed by size selection using magnetic beads; end repair and A-tailing; adapter ligation followed by magnetic bead-based cleanup of adapter-ligated DNA; PCR and cleanup of the product; quality control; denaturation; single strand circularization; enzymatic digestion; cleanup of enzymatic digestion product; quality control by Qubit dsDNA HS Assay Kit on the Qubit^®^ Fluorometer 1.0 (Invitrogen Co.), and Agilent High Sensitivity DNA Kit (Agilent Technologies) on the Agilent 2100 Bioanalyzer (Agilent Technologies). For metagenomic analysis, each experimental unit was represented by one randomly chosen animal, as some experimental units contained only one animal at the end of the experiment, and the microbiome generally was shared between animals in the same cage, indicated by sequencing all of the same cage’s microbiome samples for some cages (**Supplementary Figure 2**).

Pooled, circularized and barcoded libraries were used as templates for DNA nanoball preparation (16 samples per lane) and further analyzed on DNBSEQ-G400RS next generation sequencing platform (MGI Tech Co., Ltd.) using DNBSEQ-G400RS High-throughput Sequencing Set (FCL PE100) (MGI Tech Co., Ltd.) according to manufacturer’s instructions. Sequencing depth was calculated to achieve at least 20 million paired-end 100 bp reads per sample.

### Data Analysis

Read quality evaluation was performed with FastQC (21)⁠. Adapter clipping was performed with cutadapt v1.16 (22)⁠. Reads were trimmed from 5’ and 3’ end using 5 bp window with quality threshold 20 using Trimmomatic v0.39 (23)⁠⁠. Paired reads with length 75 bp or longer were retained for further data processing.

Reads originating from the host were removed by mapping reads against mouse reference genome GRCm38 release 96. Taxonomic classification of unmapped reads was performed with Kraken 2.0.8-beta against progenomes database (24)⁠ with additionally included mouse (GRCm38) and human (GRCh38) reference genomes (25)⁠⁠. Only reads with a confidence score of 0.5 or higher were regarded as classified. Abundance reestimation was done with bracken v2.5 at species level (26)⁠⁠. Reads classified as *Homo sapiens* or *Mus musculus* were removed from subsequent analyses. Taxonomies with low read counts were removed using filterByExpr function implemented in edgeR 3.26.8 (27)⁠.

Due to the complex experimental design, the differential abundance analysis was not performed using statistical tools that take into account compositional nature of the data. Differential abundance testing was performed with limma 3.40.6 using voom transformation with sample quality weights (28)⁠⁠. Differential testing was performed for combinations of multiple factors: metformin usage, time, diet, and sex. Correction for multiple testing was implemented with Benjamini-Hochberg method. Taxa with FDR ≤ 0.05 were regarded statistically significant. The effect of the individual mouse was accounted for using the duplicateCorrelation function in limma.

Alpha diversity was expressed as the exponential of Shannon diversity index resulting in the effective number of species, genera, and phyla at the respective taxonomic levels.

To account for the compositional nature of taxonomic data, the imputation of zero values was performed with Bayesian-multiplicative replacement method as implemented in R 3.6.3 package zCompositions 1.3.4 with default parameters (29)⁠. Resulting taxonomic data were subsequently transformed using centered log ratio transformation with scikit-bio 0.5.5 (30)⁠.

Aitchison’s distance was used as a beta diversity metric. Principal component analysis biplot was constructed from the transformed compositions with scikit-learn 0.22.

Functional analysis was performed by mapping sequencing reads against protein database and annotating matches with functional information of the corresponding protein. Paired-end reads were merged with FLASH v1.2.11. Merged and uncombined read-pairs were aligned against the SWISS-PROT database (release 2020_04) with DIAMOND (version 2.0.4). At least 80 % of the read had to be aligned with 80 % identity for it to be regarded as a possible hit. For merged read-pairs, a single best hit was retained (using DIAMOND’s option –max-target-seqs=1). Uncombined read-pairs were mapped independently. For each read up to 25 best hits were reported. If there was an overlap of matched UniProt IDs for a read-pair, then the hit with highest bit-score sum was selected from overlapping IDs. Otherwise hit with the highest bit-score from any read in a pair was selected. In both cases multiple best hits were resolved by random selection. Where possible, KEGG orthologies corresponding to UniProt identifiers were used to map KEGG BRITE functional hierarchies to UniProt IDs. Reads assigned to functional hierarchies of carbohydrate metabolism (ID 09101), lipid metabolism (ID 09103) and amino acid metabolism (09105) were counted resulting in a read count per BRITE table. BRITE IDs with median less than 100 reads were removed and differential abundance testing was performed as described in differential abundance analysis of taxonomies.

Differences in body weights and biochemical parameters between HFD-fed and CD-fed groups were determined by one-way anova and t-test. The normality of measurement distributions was assessed by Shapiro-Wilk test, equality of variances was evaluated by an F-test.

Hypothesis testing for changes in alpha diversity before and after therapy was performed using paired t-test for each metformin and diet group separately. The normality of the diversity differences between time points was assessed with the Shapiro-Wilk test. P-values < 0.05 were considered statistically significant.

## Results

### The Effect of a High-Fat Diet on Body Weight and Metabolic Parameters of Mice

**Figure 1:**
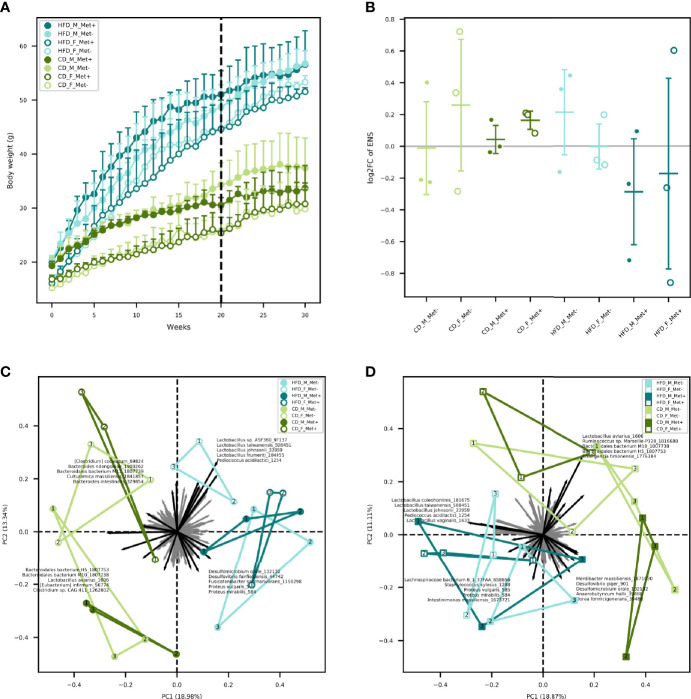
Body weight and microbiome diversity analysis. **(A)** Mean body weight of each of the experimental groups in each of the weeks. Dashed line indicates the beginning of metformin treatment. **(B)** Changes in alpha diversity in each of the groups expressed as an effective number of species. **(C)** Beta diversity in each of the groups before the beginning of metformin treatment. **(D)** Beta diversity in each of the groups after 10 weeks long metformin treatment. Samples representing each experimental unit in each of the experimental groups are shown as dots.

Mice were fed with HFD for 20 weeks in order to induce T2D manifestations. Significant differences in body weights between HFD-fed and CD-fed mice were observed after two weeks with mean body weight 22.89 ± 3.36 and 19.9 ± 3.08 g, respectively (P-value = 0.03) (**Figure 1A**). As expected, body weight was higher in males than in females in each of the diet groups. Metformin treatment had no significant effect on body weight gain in any of the studied groups.

Fasting blood glucose level was monitored by glucometer fortnightly. Upon detecting statistically significant differences in blood glucose levels between HFD-fed and CD-fed groups, we initiated regular determination of fasting glucose and insulin levels in plasma samples (**Table 1**). We calculated the HOMA-IR index, and values above two were considered to correspond to insulin resistance suggesting the onset of T2D. Before the beginning of the metformin treatment mean HOMA-IR index was above 2 in all of the HFD-fed groups but below 2 in all of the CD-fed groups. After the ten-week-long metformin treatment, fasting glucose and insulin levels in plasma samples were remeasured.

**Table 1:** Biochemical parameters before and after metformin treatment.

Group		Before treatment			After treatment		
		Glucose, mmol/L	Insulin, pmol/L	HOMA-IR	Glucose, mmol/L	Insulin, pmol/L	HOMA-IR
**HFD_M_Met-**	Mean	12.21	730.14	7.37	11.11	2079.39	17.91
	SD	1.68	449.58	4.79	4.04	385.61	9.95
**HFD_F_Met-**	Mean	12.17	309.25	2.89	14.06	661.08	6.71
	SD	1.66	147.92	1.19	1.63	250.64	1.82
**HFD_M_Met+**	Mean	15.01	1537.10	18.31	11.77	1666.22	14.33
	SD	0.80	526.92	5.92	2.81	525.32	4.59
**HFD_F_Met+**	Mean	11.46	457.83	4.06	12.00	613.48	5.35
	SD	1.50	256.10	1.91	1.18	358.59	2.76
**CD_M_Met-**	Mean	11.69	127.82	1.21	12.03	194.80	1.68
	SD	2.89	48.07	0.51	1.01	162.22	1.27
**CD_F_Met-**	Mean	8.81	96.50	0.67	10.04	127.33	1.01
	SD	1.73	10.09	0.07	2.25	57.42	0.59
**CD_M_Met+**	Mean	10.15	221.05	1.71	9.23	133.04	0.93
	SD	1.69	234.81	1.75	0.51	131.09	0.96
**CD_F_Met+**	Mean	8.29	124.45	0.78	9.43	122.71	0.86
	SD	2.24	46.60	0.21	0.25	14.93	0.09
	**Group**	**	*	*	*	***	***
	**Treatment**	NS	NS	NS	NS	NS	NS
	**Diet**	**	**	**	*	***	***
	**Sex**	*	NS	*	NS	*	NS
	**Block**	NS	NS	NS	NS	NS	NS

N = 3 in each of the studied groups. Significance codes: 0 (***), 0.001 (**), 0.01 (*), >0.05 (NS).

### Differences in Microbiome Composition Between Experimental Groups

We determined the microbiome composition of fecal samples by shotgun metagenomic sequencing. The median value of the obtained paired-end reads was 40044941 (IQR 10957336). After quality trimming and host removal, a median of 28807601 (IQR 9664060) reads were retained. The median percentage of classified reads after taxonomic classification was 40.33% (IQR 12.24%).

**Figure 2:**
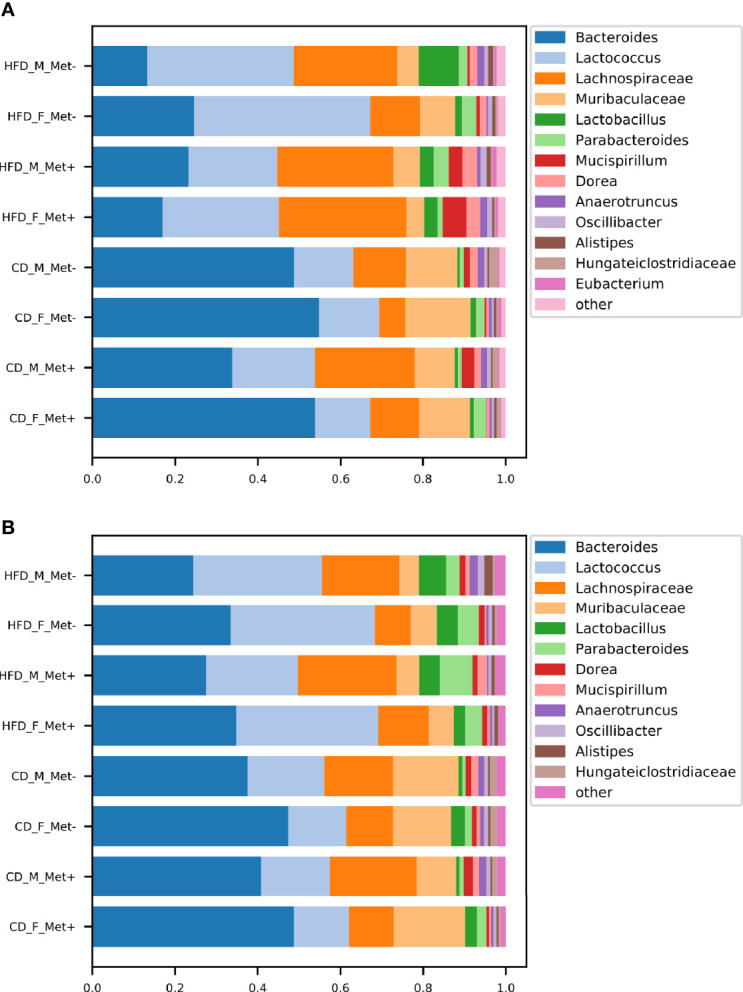
Microbiome composition at genus level in each of the experimental groups before and after the treatment. The abundances of top genera are expressed as proportions, only the genera or other lowest identified taxa with the relative proportion of at least 1% are shown. **(A)** time point before the treatment; **(B)** time point after the treatment.

The relative abundances of the top genera in each of the experimental groups are depicted in **Figure 2**. For comprehensibility reasons, only the genera with a relative abundance of at least 1% are presented. Before the initiation of the treatment, in all HFD-fed groups, *Lactococcus, Bacteroides*, and genera representing *Lachnospiraceae* dominated the gut microbiome composition. Other top taxa represented in all HFD-groups were *Muribaculaceae*, *Lactobacillus*, *Parabacteroides*, *Mucispirillum*, and *Dorea*. *Bacteroides* prevailed in all CD-fed groups, followed by *Lactococcus*, *Lachnospiraceae*, and *Muribaculaceae*, the relative abundance of which was greater in all CD-fed groups compared to HFD-fed mice.

At the time point after the treatment, we identified an increase in *Bacteroides* relative abundance in all HFD-fed groups, which was maintained at the expense of reduced relative abundance of *Lachnospiraceae*, whereas *Bacteroides*, *Lactococcus*, and *Lachnospiraceae* remained top taxa in all HFD-fed groups. All CD-fed groups were dominated by *Bacteroides*, followed by smaller proportions of *Lactococcus*, *Lachnospiraceae*, and *Muribaculaceae*.

### Diversity Analysis

#### Alpha Diversity

We identified a trend of decrease in the alpha diversity of the HFD_Met+ groups before and after metformin treatment (**Figure 1B**), though not statistically significant. In CD_Met+ groups and groups that did not receive metformin, no alpha diversity changes were observed before and after metformin treatment. Overall the effective number of species (ENS) was higher in HFD-fed groups – mean ENS before treatment was 10.99 ± 3.04 and 9.62 ± 2.64 in HFD-fed and CD-fed mice, respectively. The same was observed at the time point after the treatment – mean ENS was 10.62 ± 3.55 and 10.18 ± 1.70 in HFD-fed and CD-fed mice, respectively.

#### Beta Diversity

Beta diversity analysis revealed clustering depending on various factors included in the study (**Figures 1C, D**). Samples obtained from HFD-fed mice clustered apart from those of CD-fed mice at both time points, with the principal microbial identifiers being *Pediococcus acidilactici*, *Lactobacillus* spp., *Desulfomicrobium orale*, *Desulfovibrio fairfieldensis*, *Fusicatenibacter saccharivorans*, *Proteus* spp. before the beginning of metformin treatment. *Lactospiraceae bacterium 6_1_37FAA*, *Staphylococcus xylosus*, *Intestinimonas massiliensis* supplemented this list of microbial identifiers after the metformin treatment, though some species were represented by < 100 reads in any of the samples. Furthermore, when analyzing both sexes separately, before the treatment, HFD_F groups were directed toward *Lactobacillus* vectors, but HFD_M groups towards *Proteobacteria* members. In both sexes of CD-fed mice, principal identifiers were affiliated to *Bacteroidetes* and *Clostridia*, although the species were different for males and females. After the treatment, samples of HFD-fed mice tended to cluster closer, and *Clostridia* members appeared among the most characteristic taxa of these groups. In CD_F groups, representatives of *Bacteroidetes* and *Clostridia* remained the principal identifiers; however, in CD_M_Met+ group, a shift towards *Proteobacteria* was observed. Nevertheless, no apparent clustering regarding metformin treatment status was observed.

### Differentially Abundant Species Between Treatment Arms

**Figure 3:**
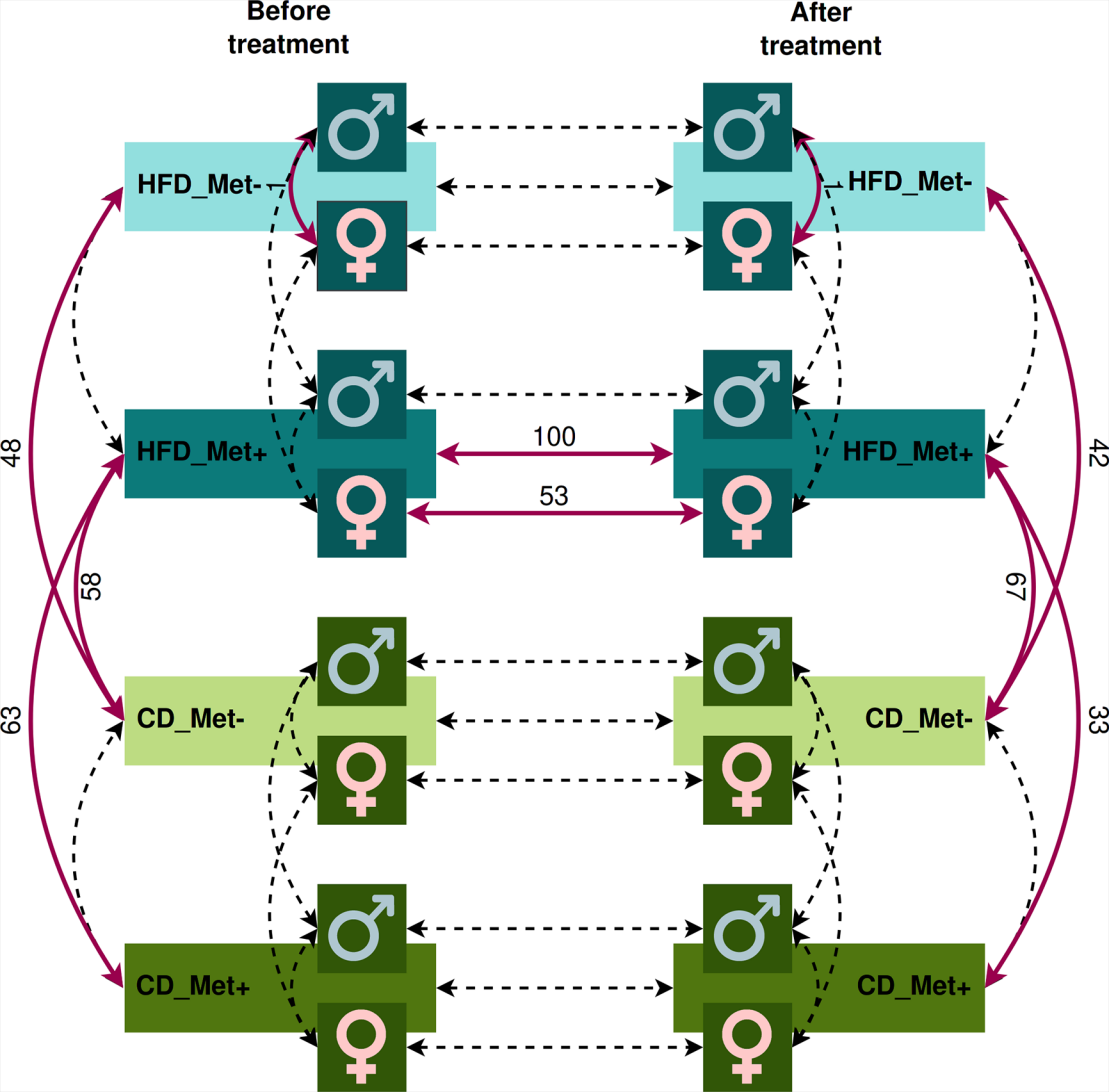
Contrasts in which microbiome compositions were compared. Dashed lines indicate the contrasts between which a comparison was performed. Red bold lines indicate the contrasts between which statistically significant differences in taxa relative abundance at species level were discovered with the numbers of the different species.

We evaluated the relative abundance of different species of microbes between groups using various contrasts shown in **Figure 3** (see **Supplementary Data** for further details).

The analysis revealed significant differences between HFD_Met- and CD_Met- groups and between HFD_Met+ and CD_Met+ groups before the beginning of metformin treatment. We observed 63 and 48 differentially abundant species between HFD- and CD-fed groups with or without metformin treatment, respectively (**Figure 3**), indicating apparent differences between these experimental units at the time point when experimental units were allocated to receive metformin treatment. At the same time, no differentially abundant taxa were identified between CD-fed groups and between HFD-fed groups. When considering the effect of sex, only one species, *Bacteroides eggerthii* (LogFC = −4.05, FDR < 0.001), was detected to be differentially abundant between HFD-fed groups, indicating that some variability may exist between identically treated groups based on sex differences. No other substantial differences in microbiome composition between the same groups before the beginning of metformin treatment were found.

We did not find any differences between HFD_Met+ and HFD_Met- groups at the time point after the treatment (**Figure 3**). The same applies to the contrast between CD_Met+ and CD_Met- groups. When each of the sexes was compared, *Bacteroides eggerthii* remained significantly differentially abundant between HFD_Met- groups (LogFC = -3.64, FDR = 0.007).

We observed a strong effect of diet on microbiome composition both before and after metformin treatment. Before initiating metformin treatment, we observed 48 and 63 differentially abundant species in contrasts between HFD_Met- and CD_Met- groups and between HFD_Met+ and CD_Met+ groups, respectively. The same applied to identical contrasts at the end of the experiment; 42 differentially abundant species were identified between HFD_Met- and CD_Met- groups and 33 – between HFD_Met+ and CD_Met+ groups. Common to all of the contrasts mentioned above, HFD was associated with a lower relative abundance of *Bacteroidales* bacteria, *Prevotella* sp., *Lactobacillus aviarius*, *Bacteroides helcogenes*, and *Bacteroides oleiciplenus*.

When comparing the differentially abundant taxa between HFD-fed and CD-fed groups representing the same metformin treatment status cross-sectionally, in all contrasts not influenced by metformin (HFD_Met- vs. CD_Met- before treatment, HFD_Met- vs. CD_Met- after treatment, and HFD_Met+ vs. CD_Met+ before treatment) in HFD groups we observed higher relative abundance of *Acetivibrio ethanolgignens* (LogFC values ranging from 3.07 to 3.84, FDR ≤ 0.008) and lower relative abundance of *Prevotella lascolai* (LogFC values ranging from -2.54 to -1.70, FDR ≤ 0.008), *Gabonia massiliensis* (LogFC values ranging from -3.09 to -1.77, FDR ≤ 0.02), *Culturomica massiliensis* (LogFC values ranging from -3.24 to -1.79, FDR ≤ 0.03), and several *Bacteroides* species (LogFC values ranging from -3.72 to -1.82, FDR ≤ 0.03).

The pairwise comparison between CD_Met+ groups before and after the treatment showed no differences in taxa relative abundance (**Figure 3**); the same was observed between CD_Met- groups.

**Figure 4:**
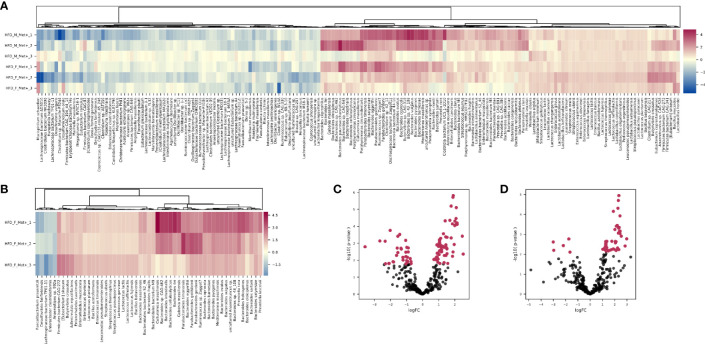
Differentially abundant species. **(A)** Heatmap showing differentially abundant species in HFD_Met+ groups before and after metformin treatment. **(B)** Heatmap showing differentially abundant species in HFD_F_Met+ group before and after metformin treatment. **(C)** Volcano plot showing P-values of differentially abundant species in HFD Met+ groups before and after metformin treatment. **(D)** Volcano plot showing P-values of differentially abundant species in HFD F_Met+ group before and after metformin treatment. Red dots represent differentially abundant species with P-values < 0.05.

Comparing HFD_Met- groups between the time points of metformin initiation and the end of the experiment, we observed no differentially abundant taxa. However, in HFD_Met+ groups, 100 species were altered (**Figures 4A, C**), with most of the species relative abundances being increased due to metformin treatment. The most pronounced changes were in the relative abundance of such *Bacteroidetes* genera as *Bacteroides*, *Parabacteroides*, *Prevotella*, *Paraprevotella*, *Porphyromonas; Firmicutes* genera *Bacillus*, *Butyrivibrio*, *Enterococcus*, *Lactobacillus*, *Lactococcus*, *Leuconostoc*, and *Streptococcus* as well as in *Enterorhabdus* representing *Actinobacteria*.

In general, the variability of the magnitude of the differences in the relative abundance of species between the samples of the studied contrast was more pronounced in species representing phylum *Bacteroidetes* (LogFC values ranging from 0.70 to 2.46). In contrast, members of the class *Bacilli – Lactobacillus*, *Lactococcus, Enterococcus, Leuconostoc, Bacillus*, and *Streptococcus* genera all representing *Firmicutes* were increased (LogFC values ranging from 0.79 to 1.17) in response to metformin in all of the samples relatively uniformly.

Analysis of relative taxa abundances in HFD_Met+ groups before and after metformin treatment in each of the sexes separately revealed sex-specific effects of metformin. In males, we did not identify significantly differentially abundant taxa while in females, the relative abundance of 53 species was significantly altered (**Figures 4B, D**). A decrease in response to metformin was observed in bacteria of *Clostridia* class including *Faecalibacterium prausnitzii* (LogFC = -2.18, FDR = 0.03), *Enterocloster clostridioformis* (LogFC = -1.74, FDR = 0.03) and *Anaerostipes* sp. *992A* (LogFC = -1.64, FDR = 0.04) as well as *Desulfovibrio fairfieldensis* (LogFC = -2.97, FDR = 0.04) of *Deltaproteobacteria* class. We identified an increase in the differentially abundant taxa in response to metformin; a subtle increase in the relative abundance of species representing *Bacilli* was observed (LogFC up to 1.36), while the increase in *Bactoroidia* class representatives was particularly pronounced, for example, *B. ilei* (LogFC = 2.85), *B. vulgatus* (LogFC = 2.45), *B. pyogenes* (LogFC = 2.43). The list of differentially abundant species in females corresponds to the taxa identified in the analysis where samples from both sexes are taken together, although the extent of changes in relative abundances was greater in females.

To further analyze the effect of metformin treatment we compared microbial compositions between CD_Met- and HFD_Met+ groups. At the time point before the treatment we observed 58 differentially abundant taxa dominated by members of *Clostridia* and *Bacteroidetes* which were increased and decreased in HFD-fed animals, respectively. *Clostridiaceae* were represented by *Hungatella hathewayi* (LogFC = 6.65, FDR < 0.001) and *Acetivibrio ethanolgignens* (LogFC = 4.30, FDR < 0.001). Members of *Lachnospiraceae* including *Roseburia* and *Dorea* followed with LogFC from 0.94 to 3.35, FDR <0.05, as well as non-*Clostridia Mucispirillum schaedleri* (LogFC = 2.89, FDR = 0.02), and *Enterorhabdus mucosicola* (LogFC = 1.27, FDR = 0.02). *Bacteroidetes* were represented by various *Bacteroidales* with the most extreme changes identified in the relative abundance of *Bacteroidales bacterium M10* (LogFC = -10.33, FDR < 0.001), in addition *Parabacteroides timonensis* (LogFC = -2.04, FDR = 0.01), members of *Prevotella* (LogFC from -2.53 to -3.61, FDR < 0.001) and others were decreased.

The same contrast at the time point after the metformin treatment revealed 67 differentially abundant species. *Bacilli* and *Bacteroides* prevailed among the increased taxa, while *Clostridia* and different *Bacteroidetes* represented decreased taxa. *Bacilli* were represented by *Staphylococcus xylosus* (LogFC = 6.40, FDR = 0.02) and members of genera *Enterococcus*, *Streptococcus*, *Leuconostoc*, and *Lactococcus* (LogFC from 1.08 to 1.58, FDR > 0.05). LogFC of *Actinobacteria Enterorhabdus mucosicola* was 1.91, FDR = 0.004). Among increased *Bacteroides* were such species as *B. salyersiae*, *B. pyogenes*, *B. vulgatus*, *B. ovatus*, *B. coprocola*, *B. eggerthii*, *B. clarus*, *B. congonensis*, *B. caccae*, and *B. acidifaciens* (LogFC from 1.12 to 2.40, FDR < 0.05). Decreased *Bacteroidetes* included *Bacteroidales bacterium M10* (LogFC = -9.68, FDR < 0.001), other *Bacteroidales*, members of *Prevotella*, *Porphyromonas*, and *Bacteroides* species *B. ilei, B. helcogenes, B. gallinarum, B oleiciplenus, B. cellulosilyticus, B. togonis, B. intestinalis*, and *B. coprophilus* (LogFC from -1.19 to -5.62, FDR < 0.05). *Clostridia* were represented by *Dorea formicigenerans* (LogFC = -3.38, FDR = 0.04) and *[Eubacterium] infirmum* (LogFC = -4.65, FDR = 0.009).

The 58 % and 76 % of differentially abundant species were shared between contrasts HFD_Met- vs. CD_Met- and HFD_Met+ vs. CD_Met+, respectively. When the same contrasts were analyzed at the time point after the treatment, 83 % and 100 % were shared.

### Differentially Abundant Functional Hierarchies Between Treatment Arms

To investigate the changes in the functional profile of the gut microbiome in response to metformin treatment, we evaluated the relative abundance of each Kyoto Encyclopedia of Genes and Genomes (KEGG) BRITE hierarchy in metagenomic data focusing on carbohydrate, lipid, and amino acid metabolism. Statistical analysis of the differentially abundant functional hierarchies between experimental groups in various contrasts corresponding to the ones indicated in **Figure 3** (see **Supplementary Data** for further details) showed significant differences in ten occasions mainly shared with the previously identified significant differences in taxa relative abundances.

**Figure 5:**
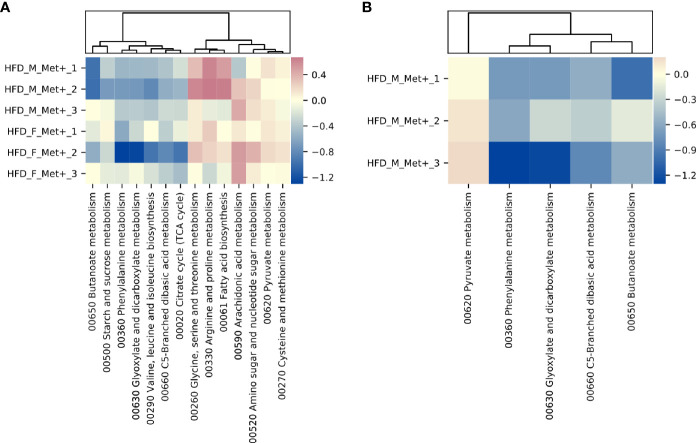
Differentially abundant KEGG BRITE hierarchies. **(A)** Heatmap showing differentially abundant hierarchies in HFD_Met+ groups before and after metformin treatment. **(B)** Heatmap showing differentially abundant hierarchies in HFD_F_Met+ group before and after metformin treatment.

In HFD_Met+ groups between the time points of metformin treatment initiation and the end of the experiment, 14 metabolic hierarchies were altered (**Figure 5A**). The most markedly decreased functions were phenylalanine metabolism (ko00360) (LogFC -0.69, FDR < 0.001), and glyoxylate and dicarboxylate metabolism (ko00630) (LogFC = -0.66, FDR = 0.002). The top increased hierarchies were arachidonic acid metabolism (ko00590) (LogFC = 0.36, FDR < 0.05), and arginine and proline metabolism (ko00330) (LogFC = 0.32, FDR < 0.05).

Similar to what was observed in the longitudinal contrast between HFD_Met+ groups when analyzing differentially abundant species, functional analysis in each of the sexes separately reinforced sex-related differences. In males, none of the analyzed hierarchies showed alterations in the number of corresponding reads. In females, 5 functions were significantly differentially represented by the reads (**Figure 5B**). The list of altered hierarchies in females coincides with the ones identified in the combined analysis of both sexes, albeit the changes were more pronounced in female mice, for example, phenylalanine metabolism (ko00360) (LogFC =1.07, FDR < 0.001), and glyoxylate and dicarboxylate metabolism (ko00630) (LogFC = -0.96, FDR = 0.003). In contrast to the combined analysis in both sexes, in females, the relative abundance of only one hierarchy - pyruvate metabolism (ko00620) was slightly increased (LogFC = 0.15, FDR < 0.05).

## Discussion

An effect of metformin on gut microbiome composition and function has been reported in previous studies both in humans and animal models and even *in vitro*. However, all rodent studies used 16S rRNA gene sequencing, and most were limited to evaluating metformin effects on only one of the animal sexes. Our study has several strengths. First, we utilized a shotgun metagenomic sequencing approach, which allows a species- or even strain-level precision in detecting microbiome representatives. Additionally, it enables the determination of the functional profile. Second, we included mice of both sexes in contrast to almost all existing studies in this specific field, providing valuable information on sex-related differences in response to metformin treatment regarding changes in the gut microbiome. Next, we employed a randomized block factorial design, which rendered the study feasible in terms of time and available resources, increased power and reproducibility, and limited the impact of various controllable and uncontrollable sources of bias. We aimed to reliably evaluate metformin’s effect on the microbiome rather than reach the maximal effect of metformin response with elevated doses. Therefore, we chose to use a smaller metformin dose (50 mg/kg body mass/day) compared to other studies (200-300 mg/kg body mass/day) to match the concentrations applied to humans. Besides, we fed the mice representing control groups in our study with a well-matched diet and not the regular chow, which is not standardized and has beneficial effects on the gut microbiome *per se* due to its short-chain fatty acid-increasing nutritional composition (31)⁠. The use of regular chow is common in previous studies assessing the effect of metformin on the microbiome, which can substantially alter the results.

This study provides valuable information on how the metformin treatment influences gut microbiome composition and functions in a mouse model with HFD-induced T2D manifestations of both sexes by using the shotgun metagenomic approach - a combination not implemented in any other previously reported study. The results of our study are in line with previous research in this area. However, we found substantial sex-related differences in response to metformin treatment. In contrast to another study involving both sexes of mice where serum glucose level decreased after metformin treatment, especially in female mice, we observed an increase in plasma glucose level in females although male mice responded with a decrease in plasma glucose level in both HFD- and CD-fed groups (18)⁠. Also, insulin resistance indicated by the HOMA-IR index declined after the metformin intervention in HFD_M_Met+ group but, inconsistent with previously reported data, insulin resistance was augmented in HFD_F_Met+ group. The same pattern was manifested in CD_Met+ groups, thereby strengthening the discrepancies in metformin effects between sexes. These differences might be explained by the fact that we used a substantially lower dose of metformin (50 mg/kg body mass/day), which is considered the maximum dose that the body can utilize efficiently (32)⁠⁠.

In addition, several studies, which have investigated sex-related differences in the gut microbiome in the context of changed diet, have identified hormonal effects on gut microbiome composition (33–35)⁠, specifically emphasizing estrogen-induced gut microbiome changes and protection against metabolic syndrome in mice, both of the C57BL/6 and *ob/ob* background. These effects possibly also contribute to the identified differences between sexes.

Intrinsic microbial diversity was higher in HFD-fed groups than CD-fed groups and decreased in response to metformin treatment in HFD-fed groups of both sexes but not in CD-fed groups in line with previous research (18)⁠. Beta diversity analysis revealed apparent clustering depending on diet and sex. The inclination toward *Lactobacillus* and *Proteobacteria* for HFD-fed female and male mice, respectively, at the time point before the treatment, coincides with the significant differences in glucose level and HOMA-IR between sexes at the same time point. This finding suggests that the relative abundance of *Proteobacteria* is associated with a higher level of insulin resistance, while *Lactobacillus* is negatively correlated with HOMA-IR.

Paired tests are more powerful in detecting microbial composition changes, explaining the fact that we observed 100 differentially abundant species comparing the samples before and after metformin treatment in HFD-fed groups. In contrast, we did not find significantly different taxa in the cross-sectional analysis comparing metformin-treated vs. untreated groups of HFD-fed mice at the time point after metformin treatment.

We observed that metformin influences the relative abundance of two *Firmicutes* classes in opposite directions, *Clostridia* being decreased or *Bacilli* being increased after metformin treatment. The relative abundance of *Bacteroidetes* and *Actinobacteria* was elevated, while all the members of *Proteobacteria* subsided in response to metformin.

We observed an increase in *Adlercreutzia equolifaciens* in both sexes, similarly to previously identified significant elevation of *Adlercreutzia* species in the fecal samples after metformin treatment. This increase has been explained by *Adlercreutzia*-mediated soybean isoflavonoid conversion to equol, which regulates glucose uptake in adipocytes by modulating known insulin-stimulation pathways (9)⁠.

Analysis of differences in microbiome composition between male and female mice showed divergence in taxonomic abundances changes. Similar to what has been reported previously, we observed a more pronounced increase in *Bacteroides* in female mice than male mice in HFD_Met+ groups (18)⁠⁠. Genera representing *Firmicutes* were more consistent between both sexes, and in subjects, their relative abundance increased in response to metformin.

We compared CD_Met- and HFD_Met+ groups to investigate whether metformin shifts microbial composition in the direction of healthy, undisturbed microbiome expected to be observed in CD-fed groups not receiving metformin treatment. Metformin increased the relative abundance of *Bacilli* and some *Bacteroidetes*, in addition to a decrease of *Clostridia*. This finding supports previous reports that metformin shifts the dysbiotic microbiome associated with metabolic disease phenotypes to a microbiome corresponding to a more healthy state by increasing the relative abundance of members of *Bacteroidetes* (36)⁠.

### Species With Decreased Relative Abundance in Response to Metformin Treatment

Species with the most substantially decreased relative abundance in response to metformin treatment *Hungatella hathewayi* (LogFC = -3.58, FDR = 0.01) has been previously described as a strictly anoxic, Gram-positive, spore-forming, rod-shaped bacterium isolated from human feces (37)⁠⁠. The bacterium used carbohydrates as fermentable substrates, producing acetate, ethanol, carbon dioxide, and hydrogen as the major glucose metabolism products.

Members of *Proteobacteria* – sulfate-reducing bacteria *Desulfovibrio fairfieldensis*(LogFC = -2.66, FDR = 0.008), *Desulfomicrobium orale* (LogFC = -2.08, FDR < 0.05) and *Mailhella massiliensis* (LogFC = -1.81, FDR = 0.04) and *Campylobacter lanienae*, previously isolated from fecal samples of asymptomatic individuals (LogFC = -2.10, FDR < 0.05) (38)⁠⁠ were also highly affected by metformin. *S*ulfate-reducing bacteria have been associated with inflammatory bowel diseases (IBD) (39)⁠⁠. It has been shown that metformin attenuates IBD severity and reduces inflammation (40)⁠, suggesting a possible role of the microbiome in metformin effects on IBD.

Our study revealed a consistent effect of metformin on the abundances of species representing *Clostridia*, more specifically members of *Lachnospiraceae* and *Ruminococcaceae*. Thus all of the identified differentially abundant taxa from these dominant butyrate-producing families (except for *Butyrivibrio* and *Rumonicoccus* sp. *Zagget7*) were decreased after the treatment in the HFD_Met+ group. Dominant butyrate-producing bacteria *Roseburia hominis*, *Roseburia intestinalis*, *Faecalibacterium prausnitzii* found in the human intestine (41)⁠, *Intestinimonas butyriciproducens* and *Eubacterium plexicaudatum* first found in mouse intestine (42, 43)⁠⁠ and *Anaerotruncus* spp. are among these taxa. Butyrate has been demonstrated to positively impact gastrointestinal tract homeostasis promoting the growth of intestinal epithelial cells, increasing the expression of tight junction proteins, and acting as an anti-inflammatory agent (44). Previous studies have reported that obesity and T2D are associated with a reduction of butyrate-producing bacteria and an increase in opportunistic pathogens (7, 45)⁠⁠. A recent randomized pilot study in which the impact of probiotic supplement on metformin’s effect on glycaemia in prediabetic subjects was assessed, has identified an increase in the relative abundance of *Anaerotruncus colihominis (Cluster IV)* only in participants receiving both metformin and the probiotic but not in participants taking either metformin or probiotic alone (46)⁠⁠. Our results show that metformin alone can impact the relative abundance of different *Anaerotruncus* species in opposite directions; *Anaerotruncus* sp. G3(2012) was decreased, while the relative abundance of uncultured *Anaerotruncus* sp. was augmented. Another member of *Lachnospiraceae – Dorea* is shown to be increased in T2D individuals and negatively correlated with the abundance of butyrate-producing bacteria (47)⁠⁠⁠. Gene-targeted approaches to investigate the butyrate-producing bacterial communities of the human gut microbiome have suggested that butyrate-producing colon bacteria form a functional group rather than a monophyletic group (48)⁠. This finding suggests that the functional niche of *R. hominis*, *R. intestinalis*, *I. Butyriciproducens*, and *F. prausnitzii* as butyrate-producing bacteria is substituted by other taxa, possibly butyrate-producing species identified with an increased relative abundance in response to metformin treatment which was discovered in our study.

A recent study has discovered *Lachnoclostridium* sp. members as fecal bacterial markers for early detection of colorectal cancer by being significantly enriched in adenoma patients (49)⁠⁠⁠. Another species with decreased relative abundance in response to metformin is *Flavonifractor plautii*, a flavonoid-degrading bacteria that have been associated with colorectal cancer in Indian patients (50)⁠. Flavonoids are substantial components of the human diet and have favorable effects on the prevention of T2D (51, 52)⁠.Recent research shows that metformin has benefits in lowering the risk of developing cancer, including colorectal cancer (53)⁠. Our findings suggest that chemoprevention effects of metformin could be mediated through the gut microbiome.

### Species With Increased Relative Abundance in Response to Metformin Treatment

This study revealed a uniform effect of metformin treatment on bacteria representing *Bacilli*. Genus *Lactobacillus* is represented *L. murinus*, *L. farraginis*, *L. paracasei*, *L. kefiranofaciens*. This finding is consistent with previous work showing that metformin increases the relative abundance of genus *Lactobacillus* in HFD-fed rats (54)⁠⁠. Probiotic supplementation with *Lactobacillus* improves glucose parameters in diabetic rats and prevents insulin resistance and hyperglycemia in HFD-fed mice (54)⁠, which is in line with our observations. *Lactococcus*, another genus of lactic acid bacteria *Lactococcus*, including *L. raffinolactis*, *L. garvieae*, *L. fujiensis*, *L. chungangensis, L. lactis, L. plantarum* is increased in response to metformin treatment, which is in agreement with a recent study evaluating the effect of metformin on the microbiome in the short term HFD-induced obesity (14)⁠.

Similar to the identified changes in *Streptococcus* in response to gut microbiome modification by HFD and metformin (14)⁠, we observed a consistent increase in several species representing this genus in HFD_Met+ groups of both sexes. Heat-killed *S. thermophilus* has been shown to moderate insulin resistance and glucose intolerance and protect the intestinal barrier in the ZDF T2D rat model (55)⁠⁠.

One of the gut microbiome’s main roles is to break down the dietary fiber and starch incompletely hydrolyzed by the host. Short-chain fatty acids (SCFA), including propionate and butyrate, are the main fermentation products of fiber digestion and can be used for lipid or glucose *de novo* synthesis. Changes in the SCFA profiles are associated with changes in the gut microbiome (56)⁠⁠. In our study, taxa associated with SCFA production, such as *Bacteroides* and *Enterorhabdus*, which convert amino acid derivatives as the energy source (57)⁠⁠, are represented among the species that are increased after metformin treatment.

*Enterorhabdus* has been represented exclusively in lean mice when compared with db/db diabetic mice (58)⁠. Moreover, Clavel et al. have identified that members of the *Coriobacteriaceae*, with the main species including *Enterorhabdus mucosicola*, *Enterorhabdus cecimuris*, in the cecum can be related to obesity resistance (57)⁠. Our data support these reports in line with previous studies that have associated body weight loss with metformin therapy (59)⁠⁠.

The relative abundance of *Bacteroides* was significantly higher after the metformin treatment, with the most represented bacteria being *B. intestinalis*, *B. vulgatus*, and *B. acidifaciens*. However, the effect was heterogeneous, as we observed a decrease of several *Bacteroides* species in some experimental units. The increased abundance of *Bacteroides* and *Parabacteroides* is in line with existing data (15)⁠. It has been described that *B. fragilis* colonization aggravates metabolic disorders induced by HFD, whereas metformin inhibits the growth of *B. fragilis* through modification of folate and methionine metabolism (60)⁠⁠. In contrast, our study has shown an increase in *B. fragilis* abundance in response to metformin treatment in HFD_Met+ groups of both sexes.

In a study on core gut bacteria of healthy mice, a high prevalence (99%) and a relatively high abundance of *Parabacteroides* was observed, suggesting that *Parabacteroides* might be essential to host health (61)⁠. This fact corresponds to metformin’s reported health benefits regarding the microbiome composition shift (8)⁠⁠. It has been hypothesized that the microbial growth-inhibitory effect of metformin is more pronounced on anaerobic organisms than aerobic organisms as anaerobic respiration produces less ATP than aerobic respiration (62)⁠.⁠⁠⁠

Metformin-induced changes in the abundance of *Akkermansia muciniphila* and metabolic improvement due to these changes have previously been shown in several studies (8, 18)⁠. Instead, our study reports no significant changes in *A. muciniphila* abundance in response to metformin in fecal metagenomic data.

### Functional Analysis

We focused the functional analysis on the hierarchies related to the three main macronutrients consumed in the diet – carbohydrates, lipids, and proteins, which can reach the colon due to their structural complexity or ingested amount, which surpasses the possibilities of primary digestion (63). Most of the altered hierarchies in response to metformin treatment in HFD-fed groups are representing amino acid metabolism-related functions. Our data show that metformin increases the metagenome functions affiliated to arginine and proline metabolism; glycine, serine, and threonine metabolism; and cysteine and methionine metabolism, in turn valine, leucine, and isoleucine biosynthesis is hindered.

In contrast to the previously reported results, in our study, metformin affects the functions associated with propionate (propanoate) and butyrate (butanoate) metabolism in opposite directions (7, 8). When HFD-fed groups were contrasted to CD-fed mice, regardless of the metformin treatment status, propionate metabolism was enriched. An analysis between HFD_Met+ groups before and after the treatment, both in the combined-sexes and female subset, highlighted a decrease in butyrate metabolism. This corresponds to a metformin-related decrease in the relative abundances of different butyrate producers representing *Lachnospiraceae* and *Clostridiaceae* observed in our study.

The role of phenylalanine-derived metabolites - phenylethylamine and trans-cinnamic acid in the context of gut microbiome functions is not well decribed (63)⁠. Study in children consuming low-phenylalanine diet due to phenylketonuria indicated that the gut microbiome of these children was depleted in butyrate-producing species and enriched in *Blautia* spp. and *Clostridium* spp. (64)⁠, which is in partial agreement to our data. We observed a significant decrease in the abundance of butyrate metabolism hierachy in HFD_Met+ groups before and after metformin treatment simultaneously with decreased phenylalanine metabolism, however, in contrast the relative abundance of *Blautia* spp. was not promoted.

Differences in experimental designs can explain discrepancies in results between our study and previously reported ones. Most of the studies have paid less attention to properly define the identified experimental unit in their experiments, which in microbiome studies most often should be a cage with animals, leading to pseudoreplication. This practice potentially evokes the exaggeration of the identified differences in data.

There are number of limitations in this study that could be addressed by future research. Although we planned and performed the animal experiment according to best practices known at the time to avoid male aggression (65)⁠, we experienced increased fighting of male mice. It could influence the results due to stress and changed social hierarchy in the cages as we implemented a humane endpoint for some animals. Hormonal effects related to cycling in female mice e.g. estrogen status, possibly influencing the gut microbiome, were not addressed in this study. Even though we performed sample size estimation before this complex experiment, this study’s results would benefit from analysis in larger groups as the variability of metabolic parameters and gut microbiome composition was noteworthy.

In conclusion, our study for the first time identified changes in microbial diversity and composition at species and strain level and functional implications of the gut microbiome in response to metformin treatment in high fat diet-fed mice. We also show that metabolic status at the time of the intervention strongly affects metformin mediated effects on microbiome. Furthermore, sex-specific differences were discovered where male mice experience more pronounced changes in metabolic markers; however, inverse effects on metabolic markers were revealed in female mice together with more marked changes in gut microbiome composition. We suggest that both sexes of animals should be included in future studies focusing on metformin effects on the gut microbiome.

## Data Availability Statement

The data presented in the study aredeposited in the European Nucleotide Archive (ENA), accession number PRJEB43173.

## Ethics Statement

The animal study was reviewed and approved by National animal welfare and ethics committee, State Food and Veterinary service, Riga, Latvia.

## Author Contributions

LS designed and performed the experiment, collected samples, prepared samples for sequencing, and wrote the manuscript. IS performed data processing and analysis. MU and IK performed the experiment. ZK, RP, and IE collected samples. JK supervised the study and revised the manuscript. All authors contributed to the article and approved the submitted version.

## Funding

This work was supported by European Regional Development Fund (ERDF), Measure 1.1.1.1 “Support for applied research” project “Investigation of interplay between multiple determinants influencing response to metformin: search for reliable predictors for efficacy of type 2 diabetes therapy” (Grant No. 1.1.1.1/16/A/091).

## Conflict of Interest

The authors declare that the research was conducted in the absence of any commercial or financial relationships that could be construed as a potential conflict of interest.

## Supplementary Material

Supplementary Figure 1 Experimental design and timeline of the study. Experiment was performed in 3 randomized blocks each separated in time. Each block contained 1 experimental unit (cage with 3 animals of the same sex) representing one of the 8 experimental groups: 1) HFD_M_Met-; 2) HFD_F_Met-; 3) HFD_M_Met+; 4) HFD_F_Met+; 5) CD_M_Met-; 6) CD_F_Met-; 7) CD_M_Met+; 8) CD_F_Met+. Abbreviations for the factors: HFD – high-fat diet-fed mice; CD – control diet-fed mice; M – male mice; F – female mice; Met- – mice not receiving metformin treatment; Met+ – mice receiving metformin treatment. After a 1 week long adaptation period experimental units were randomized into HFD- or CD-fed groups. After 20 weeks an onset of T2D manifestations was observed, first collection of fecal samples performed and each of the groups was randomized into Met- and Met+ groups for the duration of 10 weeks which was followed by the endpoint of the study and second collection of fecal samples.

Supplementary Figure 2 Gut microbiome composition in each of the mice of the same cage at time points before and after metformin treatment. Data shown for three different cages. Only the species with a relative abundance of at least 1% are shown. **(A)** Cage representing CD_M_Met+ group before treatment. **(B)** Cage representing CD_M_Met+ group after treatment. **(C)** Cage representing HFD_F_Met- group before treatment. **(D)** Cage representing HFD_F_Met- group after treatment. **(E)** Cage representing CD_F_Met- group before treatment. **(F)** Cage representing CD_F_Met- group after treatment.
