# Supplementary figures and images for: Metformin Strongly Affects Gut Microbiome Composition in High-Fat Diet-Induced Type 2 Diabetes Mouse Model of Both Sexes
Source: Front Endocrinol (Lausanne). 2021 Mar 19;12:626359. doi: 10.3389/fendo.2021.626359 (PMC8018580; doi:10.3389/fendo.2021.626359)

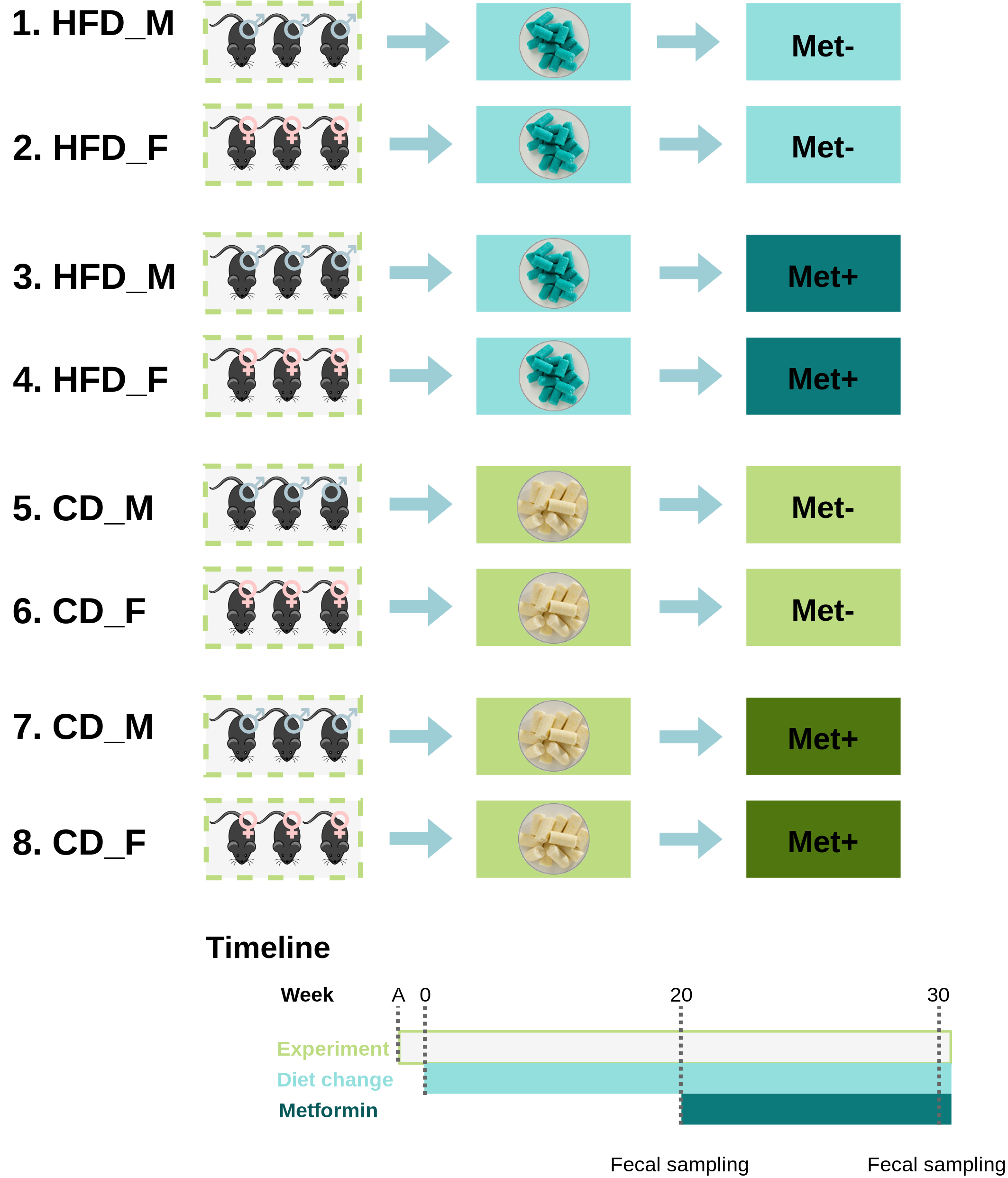

Supplement: Supplementary Figure 1 — Experimental design and timeline of the study. Experiment was performed in 3 randomized blocks each separated in time. Each block contained 1 experimental unit (cage with 3 animals of the same sex) representing one of the 8 experimental groups: 1) HFD_M_Met-; 2) HFD_F_Met-; 3) HFD_M_Met+; 4) HFD_F_Met+; 5) CD_M_Met-; 6) CD_F_Met-; 7) CD_M_Met+; 8) CD_F_Met+. Abbreviations for the factors: HFD – high-fat diet-fed mice; CD – control diet-fed mice; M – male mice; F – female mice; Met- – mice not receiving metformin treatment; Met+ – mice receiving metformin treatment. After a 1 week long adaptation period experimental units were randomized into HFD- or CD-fed groups. After 20 weeks an onset of T2D manifestations was observed, first collection of fecal samples performed and each of the groups was randomized into Met- and Met+ groups for the duration of 10 weeks which was followed by the endpoint of the study and second collection of fecal samples. [file Image_1.tif]

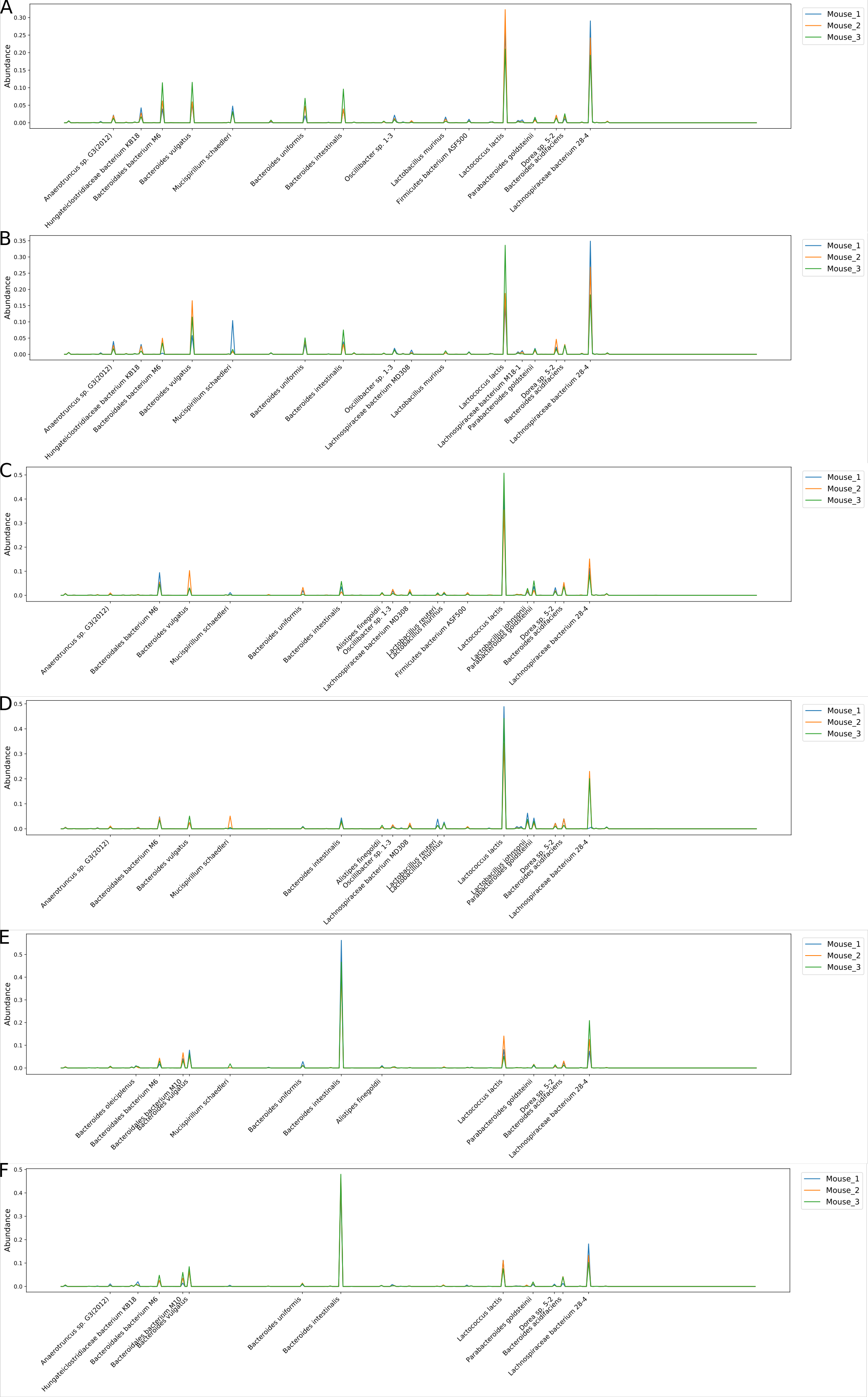

Supplement: Supplementary Figure 2 — Gut microbiome composition in each of the mice of the same cage at time points before and after metformin treatment. Data shown for three different cages. Only the species with a relative abundance of at least 1% are shown. (A) Cage representing CD_M_Met+ group before treatment. (B) Cage representing CD_M_Met+ group after treatment. (C) Cage representing HFD_F_Met- group before treatment. (D) Cage representing HFD_F_Met- group after treatment. (E) Cage representing CD_F_Met- group before treatment. (F) Cage representing CD_F_Met- group after treatment. [file Image_2.tif]
